# Current Flow Cytometric Assays for the Screening and Diagnosis of Primary HLH

**DOI:** 10.3389/fimmu.2019.01740

**Published:** 2019-07-23

**Authors:** Samuel Cern Cher Chiang, Jack J. Bleesing, Rebecca A. Marsh

**Affiliations:** ^1^Division of Bone Marrow Transplantation and Immune Deficiency, Cincinnati Children's Hospital Medical Center, Cincinnati, OH, United States; ^2^Department of Pediatrics, University of Cincinnati, Cincinnati, OH, United States

**Keywords:** flow cytometry, HLH, hemophagocytic lymphohistiocytosis, primary immunodeficiencies, clinical diagnostics, diagnostic accuracy, clinical laboratory tests, XLP

## Abstract

Advances in flow cytometry have led to greatly improved primary immunodeficiency (PID) diagnostics. This is due to the fact that patient blood cells in suspension do not require further processing for analysis by flow cytometry, and many PIDs lead to alterations in leukocyte numbers, phenotype, and function. A large portion of current PID assays can be classified as “phenotyping” assays, where absolute numbers, frequencies, and markers are investigated using specific antibodies. Inherent drawbacks of antibody technology are the main limitation to this type of testing. On the other hand, “functional” assays measure cellular responses to certain stimuli. While these latter assays are powerful tools that can be used to detect defects in entire pathways and distinguish variants of significance, it requires samples with robust viability and also skilled processing. In this review, we concentrate on hemophagocytic lymphohistiocytosis (HLH), describing the principles and accuracies of flow cytometric assays that have been proven to assist in the screening diagnosis of primary HLH.

## Introduction

Hemophagocytic lymphohistiocytosis (HLH) can be described as a systemic hyperinflammatory syndrome. It is most often thought to be caused by an inability to clear an inciting infectious or other immunologic trigger. This leads to pathologic immune activation and a positive feedback loop of ever increasing cytokine secretion and cellular cytotoxicity that ultimately results in self harm (1, 2). HLH can be classified as “primary” or “secondary” depending on whether it occurs as a result of an inborn error leading to a dysfunctional immune system like perforin deficiency, or occurs in settings such as infection, malignancy, rheumatologic, or other disease without a known underlying inherited defect in the immune system (3–5). Primary HLH can be caused by mutations in a number of genes which affect cytotoxic lymphocyte granule-mediated cytotoxicity including *PRF1, UNC13D, STX11, STXBP2, RAB27A* (Griscelli Syndrome), *AP3B1* (Hermansky-Pudlak syndrome type 2), and *LYST* (Chediak-Higashi Syndrome). Primary HLH can also include other genetic diseases such as XIAP deficiency, which is characterized by inflammasome dysregulation, and SAP deficiency which has a complicated mechanism of disease, though these diseases are usually classified as X-linked lymphoproliferative diseases (XLP) type 1 and type 2, respectively. Regardless, the classification of HLH into primary or secondary groups is sometimes difficult due to the varied phenotype presented and delays or limitations in obtaining genetic results. This has necessitated the development of faster diagnostic screening assays. Many excellent reviews exist on the subject of primary HLH and cytotoxic lymphocyte function, and the reader would be wise to refer to them for a deeper understanding on the subject (1, 6–10). In this review, we will focus on summarizing the laboratory assays currently used to screen for genetic abnormalities in primary HLH linked genes and explore their accuracy. We will also briefly discuss possible pitfalls and future directions in diagnosing diseases typically associated with HLH.

## Perforin Deficiency

NK cells and cytotoxic T lymphocytes are often grouped together as cytotoxic lymphocytes. Their primary role is to kill virus infected or malignant cells (11, 12). Perforin, the pore forming protein, is encoded by the gene *PRF1* and is a key player in this process as well as the archetypical example of primary HLH (13). *PRF1* is also historically the first primary HLH gene to be identified and is often referred to as familial hemophagocytic lymphohistiocytosis type 2 (FHL2) (14). Perforin is stored within cytotoxic granules. Once secreted from cytotoxic lymphocyte granules, perforin oligomerizes on the surface of target cells to create pores which allow the penetration of contents such as granzymes into the target. Perforin is easily stained for intracellularly in NK cells using a conjugated monoclonal antibody. Perforin has been shown to be absent or highly reduced in persons with biallelic mutations for *PRF1* gene. Staining can be performed using fresh whole blood or peripheral blood mononuclear cell (PBMC). First, the various lymphocyte lineages are extracellularly stained followed by cell fixation and permeabilization. Intracellular perforin is then stained for and the cells finally analyzed on a flow cytometer (15). To note, while freshly isolated NK cells contain perforin and are routinely used for perforin analysis, only a minority of cytotoxic T cells in “healthy” individuals express perforin. Perforin expression in resting bulk CD8^+^ cells thus varies greatly between individuals. To overcome this, *bona fide* effector T cells can be gated using CD57 if evaluation of perforin in resting T cells is desired (16, 17). This can greatly help in individuals with poor NK cell counts.

The diagnostic accuracy of perforin expression in NK cells for detecting biallelic *PRF1* mutations has recently been published and is highly accurate with sensitivity of 96.6% and specificity of 89.5% for an overall area under the curve (AUC) of 0.971 (Table 1) (18, 20). These and other reports have also shown that *PRF1* mutation carriers (a mutation in only one allele) often have clearly reduced perforin expression arguing for an allele dependent perforin expression (19, 26, 27).

**Table 1:** Sensitivity and specificity results for the diagnosis of primary HLH and related diseases extrapolated from various studies using a range of immunological assays.

**References**	**Gene(s) studied**	**Assay description**	**Sensitivity, specificity (%)**	**Number of primary cases**
Abdalgani et al. (18)	*PRF1*	Direct Intracellular staining of NK or CTL	97, 90	48
Tesi et al. (19)	*PRF1*	Direct Intracellular staining of NK or CTL	100, 100	14
	*PRF1*	NK cytotoxicity (chromium release) upon K562 stimulation	100, 95	14
Rubin et al. (20)	*PRF1*	Direct Intracellular staining of NK or CTL	97, 83	29
	*PRF1, UNC13D, STX11, STXBP2, RAB27A, LYST, AP3B1*	NK cytotoxicity (chromium release) upon K562 stimulation	60, 72	84
	*UNC13D, STX11, STXBP2, RAB27A, LYST, AP3B1*	NK degranulation (CD107a) upon K562 stimulation	94, 73	32
Bryceson et al. (21)	*UNC13D, STX11, STXBP2, RAB27A, LYST*	NK degranulation (CD107a) upon K562 stimulation	96, 88	90
Chiang et al. (16)	*UNC13D, STX11, STXBP2*	NK degranulation (CD107a) upon K562 stimulation	94, 84	16
	*UNC13D, STX11, STXBP2*	NK degranulation (CD107a) upon anti-CD16 antibody stimulation	88, 98	16
	*UNC13D, STX11, STXBP2*	CTL degranulation (CD107a) upon anti-CD3 antibody stimulation	88, 98	16
Chiang et al. (22)	*LYST*	NK degranulation (CD107a) upon K562 stimulation	85, 75	20
	*LYST*	NK degranulation (CD107a) upon anti-CD16 antibody stimulation	86, 96	21
	*LYST*	CTL degranulation (CD107a) upon anti-CD3 antibody stimulation	90, 90	20
	*LYST*	NK cytotoxicity (chromium release) upon K562 stimulation	89, 94	18
Hori et al. (23)	*UNC13D*	NK degranulation (CD107a) upon K562 stimulation	100, 71	6
	*UNC13D*	CTL degranulation (CD107a) upon anti-CD3 antibody stimulation	100, 100	6
Gifford et al. (24)	*SH2D1A*	Direct Intracellular staining of NK or CTL	87, 89	15
	*XIAP/BIRC4*	Direct Intracellular staining of NK or CTL	95, 61	19
Ammann et al. (25)	*XIAP/BIRC4*	Monocyte activation (TNF) upon L-18MDP stimulation	100, 100	12

**Figure 1:**
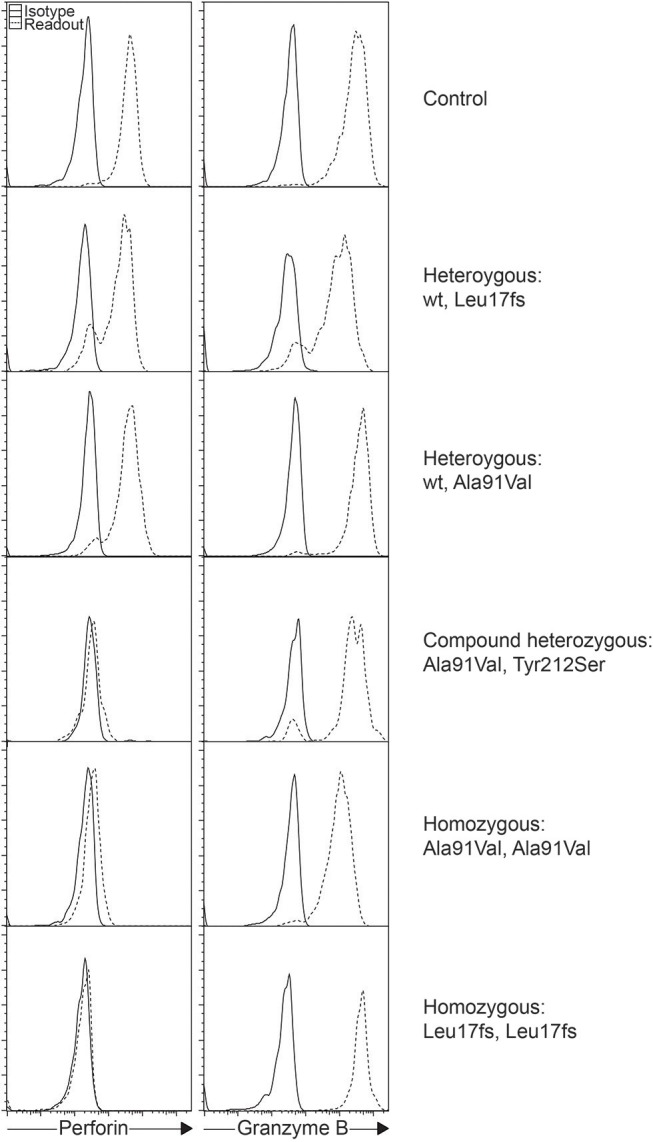
Intracellular staining of perforin and granzyme B in different individuals. Histograms represent gated NK cells showing varying levels of perforin expression as a consequence of the different *PRF1* variants as shown.

The A91V alteration in *PRF1* is unique. Having a high prevalence of 0.22 to 3.9% depending on the population studied, it has been assumed to be less pathologic (Figure 1) (28–31). However, *in vitro* studies have shown that A91V leads to reduced perforin function (32, 33). Individuals with A91V in both compound heterozygous and homozygous state can be identified by laboratory assays and show low to no residual protein expression, and such results may be indiscriminable from other pathologic *PRF1* mutations (30, 34, 35).

The lack of perforin leads to an inability to kill target cells. This functional defect can be detected by lowered chromium release using the radioactive chromium cytotoxicity assay (36). Because the chromium release assay shows suboptimal accuracy, many have turned to screening for primary HLH diseases with perforin staining coupled with the degranulation/exocytosis/CD107a assay in place of or in addition to chromium release NK cell function testing. The CD107a assay examines if cytotoxic lymphocytes (NK cells and CTL) can release secretory lysosomes as described below, but this assay does not report if target cells are killed. Samples from patients with perforin deficiency will not show any degranulation abnormalities but is nonetheless often run to confirm normal degranulation. Typical perforin deficiency can thus be confidently diagnosed based on the lack of perforin staining, deficient NK cell cytotoxicity, but normal degranulation.

## Secretory Granule Exocytosis Deficiency

Autosomal recessive mutations in *UNC13D, STX11*, or *STXBP2* have been linked to primary HLH disease. These encode the proteins Munc13-4, syntaxin-11, or Munc18-2, and as diseases are known as FHL3, FHL4, or FHL5, respectively. The proteins encoded are crucial for perforin-containing secretory lysosome exocytosis, a process more commonly referred to as degranulation. Defects in *RAB27A, LYST*, and *AP3B1*, leading to Griscelli syndrome type 2 (GS2), Chediak-Higashi syndrome (CHS), and Hermansky-Pudlak syndrome type *2* (HPS2), respectively, also cause defective degranulation. These latter patients often manifest with HLH and usually, but not always, occulocutaneous albinism (22, 37–42). Together, these 6 genes can be grouped for diagnostic screening as they show a similar cellular phenotype of failed secretory lysosome content release and failure to kill target cells.

**Figure 2:**
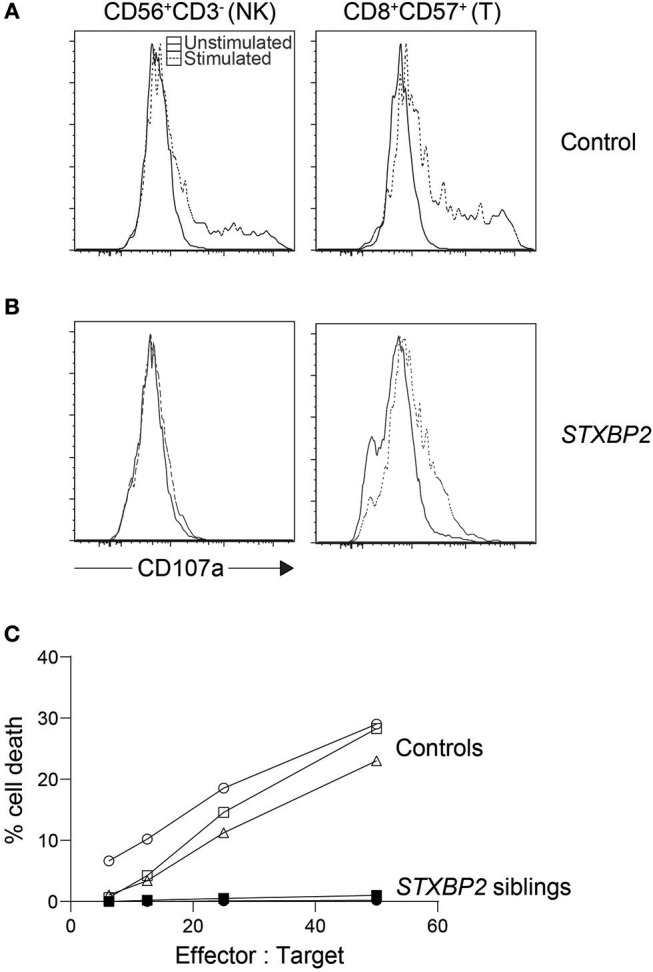
Cytotoxic lymphocyte evaluation of an *STXBP2* patient. We performed NK cytotoxicity as well as NK and T cell degranulation using fresh PBMC from a case with homozygous c.1430C>T (p.Pro477Leu) mutations. While **(A)** control NK cells and CD8^+^CD57^+^ T cells degranulated as expected when stimulated, respectively with K562 or anti-CD3 antibody, **(B)** the patient's cytotoxic lymphocytes did not. **(C)** NK cytotoxicity was also evaluated via ^51^Cr release and found deficient. In addition, we included cytotoxicity data from a sibling carrying the same homozygous mutation.

At this juncture, it is important to differentiate between the terms “NK cell degranulation” and “NK cell function,” as they are often thought to be one and the same. The NK degranulation assay, also known as CD107a or NK exocytosis assay, evaluates if CD107a containing secretory lysosomes are able to release their content and thus deposit CD107a on the external cell membrane where it is measured as a surrogate for degranulation (Figure 2). Under the microscope, CD107a and perforin often co-localize and so it is assumed that when granules bearing CD107a are externalized, perforin would also most likely be released at the immune synapse (43, 44). In the case of perforin deficiency, the CD107a assay is not useful as a screening tool because secretory lysosomes without perforin are still released and CD107a still expressed on the cell membrane. The CD107a assay is also unable to detect whether granules are headed toward the immune synapse where the target cell is being engaged. When stimulating NK cells *in vitro* with anti-CD16 antibody, the release of secretory lysosomes are non-polarized which would not be efficient for target cell elimination (43). The CD107a assay has been found useful for the diagnosis of FHL3-5, GS2, CHS, and HPS2, and possibly ORAI1, STIM1, and HPS10 (45–48), because in all these cases, secretory lysosomes are unable reach the cell membrane or fail to fuse with the cell membrane leading to the absence of surface CD107a after relevant stimulation. But, in cases of preserved detection of CD107a upregulation, additional testing to evaluate NK cell killing may be needed, as lysosome degranulation does not necessarily equate to the death of target cells.

As such, the often crowned “gold standard” chromium release assay still holds relevance since described in the 1960s (49, 50). In this assay, K562 cells (ATCC, CCL-243) first preloaded with radioactive chromium-51 will be killed by NK cells and the extent to which the stored chromium is freed is taken to represent the percentage of K562 killed (51–53). No published data exists exploring the accuracies of NK cytotoxicity assay in diagnosing each subtype of primary HLH, possibly due to sample number limitations. Only one recent study attempted to systematically quantify the accuracy of the chromium release NK cell function assay when used in the clinical lab setting for diagnosing *PRF1, UNC13D, STX11, STXBP2, RAB27A, LYST*, and *AP3B1* mutations, and found it lacking with a sensitivity of 60% and specificity of 72% (Table 1) (20).

The low accuracy of this assay, often used during acute phase HLH, may be partly blamed on the assay's dependency on the NK cell percentage in the sample. HLH patients normally experience large expansions of CD8 T cells, and stressed blood samples from these patients often leave large numbers of RBC and cell debris in the peripheral blood mononuclear cell (PBMC) suspension after ficoll. This leads to an artificially low NK cell percentage which is often unaccounted for, giving an impression of reduced NK function when in fact it is due to the overwhelming number of other cells in the mix. Because the assay is sensitive as such, care must be taken when interpreting poor NK cytotoxicity results especially during acute HLH as it could indicate poor sample quality rather than dysfunctional NK cells. While this assay has many limitations, the result distinctly demonstrates whether or not target cells are finally killed (Figure 2) (54). Numerous flow-, colorimetric-, and imaging-based cytotoxicity assays have been touted as possible chromium release assay replacements but no large cohort of primary HLH cases has been validated on any of these platforms (55–59). Pending such reports, the chromium release assay is still the only published clinical standard for NK functional studies.

Therefore, we currently rely on the CD107a NK cell degranulation assay for the screening diagnosis of primary HLH related to mutations in *UNC13D, STX11, STXBP2, RAB27A, LYST*, and *AP3B1*. The most commonly used NK degranulation assay tests rested PBMC stimulated with the myelogenous leukemia cell line K562 (21). After co-incubation for several hours, the percentage of NK cells bearing surface CD107a or the fluorescence intensity of CD107a positive NK cells is then evaluated. Persons with a defect in secretory lysosome transport or membrane fusion will show greatly reduced surface CD107a levels (Figure 2). A pan European study found 97% of FHL3-5 and 85% of GS2 and CHS cases had abnormal percentage of NK cell degranulation (<5% CD107a^+^ NK cells) to give an overall sensitivity of 96% and specificity of 88% in diagnosing a genetic degranulation disorder (Table 1) (21). A follow-up study on a North American cohort evaluated CD107a mean channel fluorescence (MCF) of NK cells instead of percentage of degranulating cells (20). It found 93.8% of patients with biallelic mutations in an HLH-associated degranulation gene with lowered CD107a MCF but only 60.4% of individuals without biallelic mutations in relevant genes with normal CD107a levels, giving an overall area under the curve of 0.86. More recently, a cohort of 21 CHS cases has likewise confirmed the CD107a assay is able to accurately identify primary defects in NK degranulation (22). In the first two studies, a sizable portion of controls were found to have lowered NK degranulation. This could be due to technical issues, stress during blood sample transport, medications leading to reduced lymphocyte reaction, or epigenetic changes resulting in NK cells with a particularly skewed functional response (60–63). So while better than the chromium release assay, the NK-K562 degranulation assay, like all diagnostic assays, is not perfect.

To overcome the shortcomings stemming from an overreliance on any single test, NK degranulation can also be evaluated through other means, for example, via stimulation using PMA, activating antibodies such as anti-CD16 targeting the Fc receptor, or activation of synergistic NK receptors (16, 64, 65). Preliminary data has found Fc stimulation induced degranulation returns 88% sensitivity and 98% specificity in a cohort of 16 FHL3-5 (Table 1) (16). We can thus infer that both NK cell natural cytotoxicity and antibody-dependent cellular cytotoxicity are defective in classical primary HLH. This is an important point to note as immunodeficiencies could affect only one specific pathway. For instance, a certain CD16 (FcγRIIIA) mutation was found to impair natural NK cytotoxicity but Fc specific function was intact (66). Current standard clinical tests limited to only K562 stimulation would be insufficient for detecting abnormalities in such cases.

Cytotoxic T lymphocytes have also been found defective in degranulation in the context of primary HLH due to mutations in the genes required for normal degranulation. Previously, T cell blasts had to be grown up over weeks in order to sufficiently stimulate perforin production in T cells and generate enough cell numbers for experimentation (21). More recently, it was noticed that specific populations of T cells, namely CD3^+^CD8^+^CD57^+^ contain perforin and granzymes *ex vivo* without prior need for stimulation (17). This population of *bone fide* effector cells, by virtue of perforin expression, was found to efficiently degranulate upon anti-CD3 antibody stimulation. Crucial to our context, when tested on primary HLH samples, CD3^+^CD8^+^CD57^+^ T cell degranulation was defective to a similar level as in NK cells (16). A small confirmatory study found high sensitivity with a cohort of biallelic pathogenic *UNC13D* variants (23). With multiple ways to induce degranulation on multiple cell types, we could speculate on possible undiscovered immunodeficiencies that affect only NK cells or T cells and detectable only with a combination of various degranulation assays.

Like perforin, it is possible to directly detect Munc13-4, syntaxin11, Munc18-2, and Rab27a with antibodies (67–69). However, this is usually performed with western blot. One exception is Munc13-4 detection in platelets with flow cytometry (70, 71). Although this assay has been found to be highly accurate for predicting *UNC13D* mutations, the antibody used is polyclonal and not commercially available.

Taken together, when primary HLH is suspected, performing the triad of perforin staining, NK and/or T cell degranulation, and NK cytotoxicity will give a more complete evaluation of cytotoxic cell activity and improve HLH diagnosis. While all the assays are individually accurate, we suggest moving toward a “multiplexing” of degranulation assays in the future to increase confidence in diagnosis, provide security should any one cell population be poorly represented, and pave the way for detecting degranulation deficiencies in specific pathways or cell types. Additionally, validating a radioactivity-free killing assay that accounts for effector cell counts would be highly useful for true assessment of cytotoxic lymphocyte function.

## X-linked Diseases

The genes *SH2D1A* and *XIAP/BIRC4* encode the proteins SAP and XIAP, respectively. Deficiencies in these proteins lead to X-linked lymphoproliferative disease type (XLP) 1 and 2 (72, 73). As their names imply, both genes are X-linked and often manifest HLH with Epstein-Barr virus (EBV) infection (74–76) but beyond that, XLP1 and XLP2 have quite different phenotypes and share little functional or structural similarities (77).

**Figure 3:**
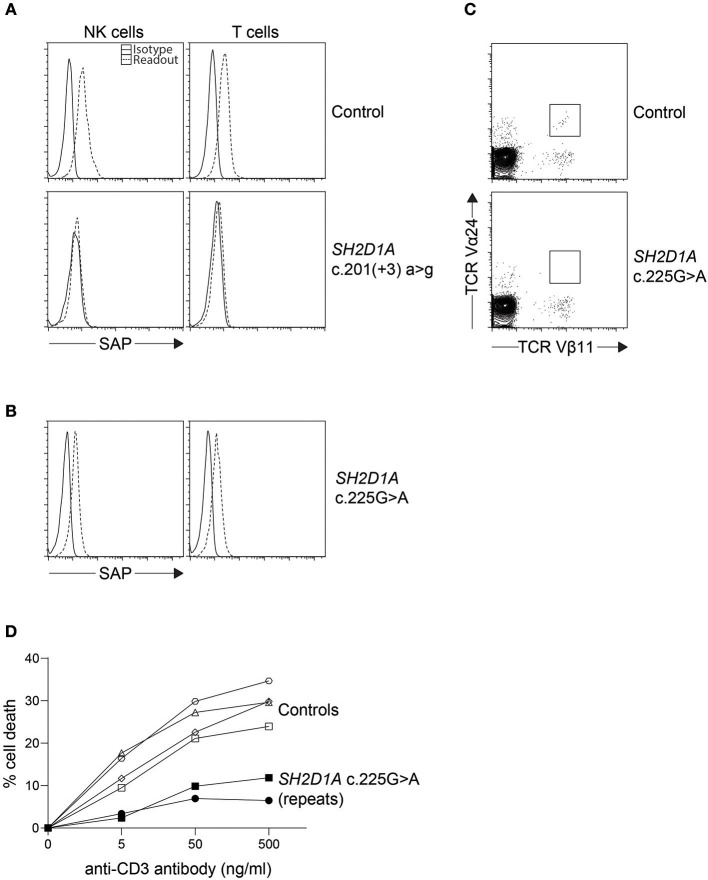
Indirect diagnosis of XLP1. **(A)** While most *SH2D1A* mutations result in absent or lowly expressing SAP protein levels, we found **(B)** a clinically suspicious patient with c.125G > A (p.Cys42Tyr) missense mutation with only a slight reduction in SAP protein expression by flow cytometry. The patient was thus further evaluated for **(C)** iNKT numbers on bulk CD3^+^ cells and **(D)** restimulation-induced cell death (RICD) via anti-CD3 antibody repeated on two occasions. The low iNKT counts and reduced cell death upon TCR restimulation provided evidence that the missense *SH2D1A* variant found was indeed pathological.

**Figure 4:**
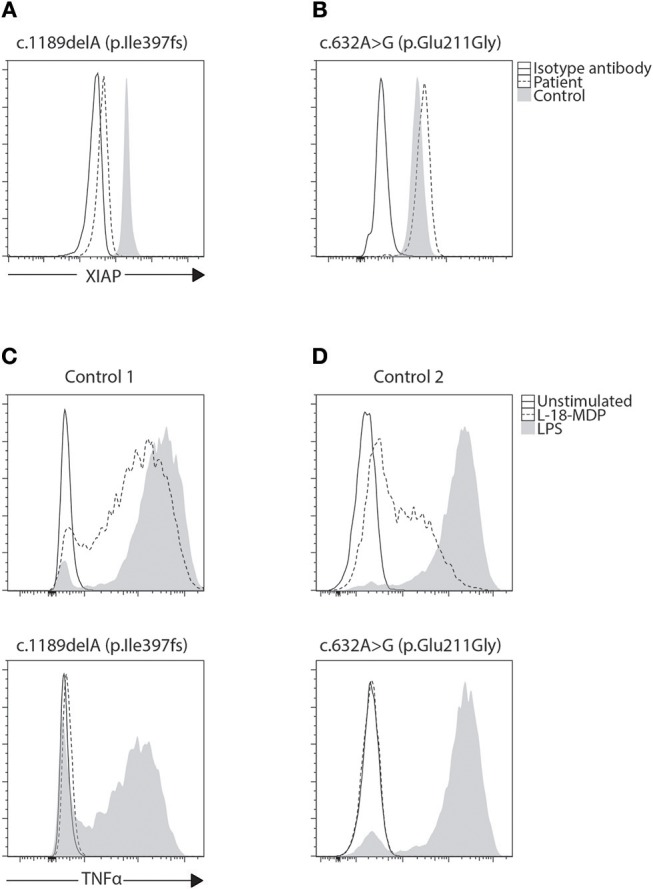
Phenotyping and functional evaluation for XLP2. **(A)** While a majority of *BIRC4* mutations present with absent or lowly expressing XIAP protein levels, we found **(B)** a patient with c.632A>G (p.Glu211Gly) variant of uncertain significance (VUCS) with only a slight reduction in XIAP protein expression by flow cytometry. Functional evaluation of XIAP can be done through stimulation of NOD2 with L18-MDP. This signaling pathway requires XIAP for TNF transcription through NF-κB. **(C,D)** When examined, both these cases show equally defective TNF production regardless of XIAP expression revealing the VUCS is in fact a damaging mutation. LPS acts as a positive control that signals through TLR4 demonstrating preserved cellular function in patient cells.

Similar to perforin, SAP and XIAP monoclonal antibodies exist and have been validated clinically for direct intracellular protein detection (Figures 3, 4) (78–80). However, care must be taken when reading such reports as certain pathologic variants have been found to preserve antibody binding leading to false negative (false normal) results (81–83). Also, while the absence of binding can be equated with the absence of that protein and thus strongly suggests a defect, the binding of an antibody to its antigen says nothing about the function of the protein bound. As such, patients expressing normal SAP and XIAP levels, or for that matter all direct antibody phenotyping tests, should still be sequenced if clinically suspicious. Bimodal staining patterns are also useful in identifying female carriers as well as estimating the level of chimerism for transplant monitoring (24, 79). For XIAP, there has been reports of non-random X inactivation in some female carriers. Lymphocytes bearing the wild-type allele have been seen selected in some while others show the opposite, skewing toward the defective X chromosome at risk for disease manifestations (73, 84, 85). Direct screening of SAP returns 87% sensitivity and 89% specificity for the prediction of pathologic mutations in *SH2D1A* while direct screening of XIAP gives 95% sensitivity and 61% specificity (Table 1) (24, 86).

It has been demonstrated that both SAP and XIAP are required for the development of normal invariant NKT (iNKT) cells and for normal T cell restimulation-induced cell death (RICD) (73, 76, 87, 88). As such, iNKT quantification and RICD assays can be performed for cases where direct staining is inconclusive, or if further supporting data is desired (Figure 3). A more sophisticated cytotoxic assay looking at inhibitory 2B4 signaling in NK cells has also been reported to discriminate functional SAP deficiency (89). Likewise, a functional test exists where XIAP function is investigated downstream of NOD2 stimulation on monocytes. Following stimulation with L18-MDP, TNF is normally produced by CD14 positive cells. However, patients with pathologic mutations in XIAP, even where XIAP protein staining was found normal or in patients with milder clinical phenotype, all had equally defective TNF production and could easily be discriminated (Figure 4) (25). A cutoff of 10% TNF-producing monocytes perfectly distinguished 12 XIAP patients from 29 healthy controls and 6 female carriers (Table 1). Subsequent reports demonstrated the assay's usefulness in diagnosing inflammatory bowel disease (IBD) cases with novel XIAP mutations (90, 91). By performing phenotyping as well as functional assays side by side, it is hoped that future cases might be more accurately identified.

## Other Primary Immunodeficienices

A host of patients with other diseases such as ALPS, CGD, CVID, and SCID, as well as variants in genes including *BTK, CARMIL2, CD27, ITK, LRBA, MAGT1, NEMO, PIK3CD, RAG2, WAS*, NLR genes, and STAT genes, have been implicated with possible HLH (92–94). The assays described so far including NK cell degranulation and cytotoxicity will be of little diagnostic use here except to rule out defective secretory lysosome transport. For some genes, there exist flow cytometric assays that can assist with diagnosis. For example T, B, and NK specific subset phenotyping panels can pick up ALPS (increased double negative T cells), X-linked agammaglobulinemia due to mutations in *BTK* (low B cell counts or BTK expression), mutations in *CD27* (absent surface expression of CD27), mutations in *MAGT1* (lowered NKG2D expression), and a variety of SCID disorders (very low B, T, and/or NK counts, reduced recent thymic emigrants and CD45RA expression) (95). The neutrophil oxidative burst assay is an excellent assay for the diagnosis of CGD (96). WAS can be accurately diagnosed through direct staining of intracellular WAS protein (97). Multiple excellent reviews exist for PID diagnostics (98, 99).

A second group of primary immunodeficiency genes demonstrate defective NK cell activity without pronounced HLH. However, before suggesting that NK degranulation and cytotoxicity assays could be used in helping with the diagnosis of these PIDs, larger cohorts of patients must be collected for evaluation to confirm and explore cytotoxic lymphocytes further including: whether or not both NK and CTL are affected, if both degranulation and cytotoxicity are defective, and if the majority of mutations in that gene share the same phenotype. Genes in this group include *AP3D1, CTSC, FERMT3, GATA2, IRF8, MYH9, ORAI1*, and *STIM1* (45, 47, 48, 100–107). From this list, we know that not all persons for whom NK cell function is defective should be labeled primary HLH. Moreover, a thorough evaluation is hampered as many of publications lack NK degranulation or cytotoxicity data, something we hope future endeavors will address. These genes are thus currently not grouped together with the “classical” primary HLH family because clinical HLH is not usually the outstanding feature. Most are also very rare leading to difficulty in performing large cohort evaluations of cytotoxic lymphocyte activity.

## The Future of HLH Diagnostics

The HLH field has come some ways since the HLH-2004 criteria were established (108). A European cohort of cases with clinical HLH and PID other than defects in cytotoxicity found 63 cases, 80% of which were CGD and CID (109). Across the Atlantic, another HLH cohort was comprised of only 19% primary HLH disorders, with 58% of patients having other PIDs including genes associated with inflammasome function (92). We reason the high percentage of “non-classical-HLH” cases is a reflection of improved HLH awareness within the community and should be looked upon positively. These and other studies looking into specific sensitivities of various HLH-2004 criteria have found them wanting (110–112). The concern often cited is the inability to distinguish between primary HLH, secondary HLH, and other PIDs. A simple solution that can easily be adopted today is increased screening. As can be concluded from Table 1, many subtypes of primary HLH can be diagnosed with good accuracy. As such, the fulfillment of HLH criteria should act as an actionable gateway to seriously consider PID by performing various laboratory tests as discussed. This in tandem with advanced sequencing should more often than not provide conclusive diagnosis for all the common primary HLH cases. As previously mentioned, we believe the field of HLH diagnostics will move toward a “multiplexing” of screening assays to more quickly screen for multiple defects simultaneously.

The evaluation of gene expression signatures is an exciting development that could help untangle some of the primary vs. secondary HLH questions going forward. Unique interferon-stimulated gene signatures have been found in systemic lupus erythematosus differentiating it from rheumatoid arthritis and control samples (113, 114). Other studies successfully used the interferon score to identify various Mendelian Type-I IFN-mediated autoinflammatory diseases (115, 116). Preliminary work to define a HLH signature has also been performed with favorable results (117, 118). While research on this area is in its infancy today, we postulate a future where specific gene expression fingerprints from tens or hundreds of genes would be elucidated for the various shades of HLH. We could then quickly and accurately segregate HLH into several subcategories as well as deduce their disease status. The signatures could not only act as a “precision” diagnostic tool but also afford us a deeper cellular mechanistic understanding on the pathobiology of various closely related diseases, and thus opportunities for “precision” therapeutics. We are excited to see what the future holds in terms of HLH diagnostics.

## Author Contributions

RM initiated the manuscript which SC wrote and JB edited.

### Conflict of Interest Statement

The authors declare that the research was conducted in the absence of any commercial or financial relationships that could be construed as a potential conflict of interest.
